# Examination of Brains in Shell-shock without Visible External Injury

**Published:** 1918-04

**Authors:** 


***Examination of Brains in Shell-shock without Visible External Injury.*** By Lt. Col. F. W. Mott, R. A. M. C. T. *Journal of the Royal Army Medical Corps*, Dec., 1917. Abs. by Cap. J. M. Wolfsohn, M. R. C.

In this paper, Mott examines the brains of two cases of death from shell-shock, without visible injury and without punctate hemorrhages indicative of gas-poisoning. His description serves to explain: 1) Sudden death in shell-shock, 2) Clinical symptoms which persist for some time after the commotion of the brain in non-fatal cases (shell-shock).

He found a generalized early chromatolytic change in the cells of the cerebral nervous system which varied in intensity. The cells most affected were the small cells in which the basophilic substance had almost disappeared. The larger cells, the Nissl granules were smaller and less closely packed than normally. The small cells of the medulla and pons were slightly swollen, the nucleus was large and clear; the same but less marked change was present in some of the large cells, which indicated a relative degree of exhaustion of the kinetoplasm.

The vessels of the pia-arachnoid part of the brain were congested, with scattered subpial hemorrhages of microscopic size almost everywhere. Congested veins, with hemorrhages into their sheaths, with occasional extravasation of blood were found in the corpus calossum, internal capsule, pons, and medulla.

Mott gives the opinions of French and German officers regarding shell-shock by " windage ".

Leri, for example, says, " True commotion appears only to be produced at the approximate distance of ten meters from large projectiles ". The mere proximity of the explosion is sufficient to cause organic changes in the brain and cord, by compression and decompression of gases, the result of explosion, and of the atmospheric air; this is apart from actual concussion caused by violent contact with solid materials.

Mott states that the vast majority of fatal cases of shell-shock are more emotional in origin than commotional and occur in subjects of an inborn neurotic or neuropathic temperament; the two conditions may, however, be associated.

The presence of blood in the spinal fluid is found in cases where organic changes have occurred.

Leri states, that in cases of commotion, patients are generally depressed, asthenic, aboulic, and often more or less confused mentally. The Germans do not accept the exclusive psychogenic role of the concussion syndrome, although some authors have quite often found labyrinthine lesions in this class of case.

Two hypotheses to explain organic lesions by commotion are given.

1. Compression of the gas and atmosphere on the brain and spine and their contents, causing a molecular disturbance in the neurones, especially in those of the nuclei in the floor of the fourth ventricle where the cerebrospinal fluid acts as a water cushion, on which the cardio-respiratory centres rest.

2. Compression is followed by a corresponding decompression, causing liberation of the bubbles of gas in the blood and tissues leading to embolism.

Both compression and decompression probably act in causing vascular disturbances, namely, arterio-capillary anemia, venous congestion and rupture of the thinner vessels, with microscopic hemorrhages.

Mott then describes two cases of death from shell-shock without visible injury, and gives excellent microscopic notes and photographs, with comments.

